# Video based object representation and classification using multiple covariance matrices

**DOI:** 10.1371/journal.pone.0176598

**Published:** 2017-06-08

**Authors:** Yurong Zhang, Quan Liu

**Affiliations:** 1 Wuhan University of Technology, School of Information Engineering, Wuhan, China; 2 HuiShang Vocational Technical College, Electronics Information Department, Hefei, China; Nanjing Normal University, CHINA

## Abstract

Video based object recognition and classification has been widely studied in computer vision and image processing area. One main issue of this task is to develop an effective representation for video. This problem can generally be formulated as image set representation. In this paper, we present a new method called Multiple Covariance Discriminative Learning (MCDL) for image set representation and classification problem. The core idea of MCDL is to represent an image set using multiple covariance matrices with each covariance matrix representing one cluster of images. Firstly, we use the Nonnegative Matrix Factorization (NMF) method to do image clustering within each image set, and then adopt Covariance Discriminative Learning on each cluster (subset) of images. At last, we adopt KLDA and nearest neighborhood classification method for image set classification. Promising experimental results on several datasets show the effectiveness of our MCDL method.

## 1 Introduction

With the recent development in imaging techniques, multiple images of an object are usually available in many cases, such as video based surveillance, multi-view camera networks, etc. Object recognition from these multiple images is formulated as an image set (video) classification problem and has attracted more and more interests and attention in computer vision and machine learning area in recent years [1, 2, 3, 4, 5, 6, 7]. This technique can be widely used in many computer vision problems. For example, in visual object search task [8,9,10], one can use multiple images to retrieve and recognize the similar visual objects. In face recognition problem [11], we can also use multiple face images to conduct person identification. Compared with the traditional single image based object recognition and learning, video model generally contains more visual appearance contents and thus performing more robustly and effectively on image set representation [12, 13, 14, 15, 16, 17, 18].

One of the main problems and challenges for video based object recognition is to develop an effective method to represent an image set or sequence. In recently years, many methods have been proposed for image set representation and classification. Other main problems include image set classifier development, image set clustering methods and so on. In this paper, we focus on image set representation. Kim et al. [19] proposed Discriminant-analysis of Canonical Correlations (DCC) to represent an image set by using single linear subspace. Hamm et al. [3] proposed Grassmann Discriminant Analysis (GDA) which uses multiple local linear subspaces to represent an image set. Besides linear subspace, nonlinear subspace methods have also been used for image set representation. For nonlinear subspace based representation, Wang et al. [20] presented an image set with nonlinear manifolds and used Manifold-Manifold Distance (MMD) method for image set representation and classification. Wang et al. [21] also proposed Manifold Discriminant Analysis (MDA) to obtain a more discriminative feature space to represent a set of images. In additional to above methods, probabilistic models have also been used for image set representation and classification. Shakhnarovich et al. [4] used a single Gaussian model for set modeling. Arandjelovic et al. [1] further provided a method to use Gaussian Mixture Models (GMM) to image set representation. Wang et al. [11] proposed Discriminant Analysis on Riemannian Manifold of Gaussian Distributions (DARG) to learn a discriminative representation for image set.

As one of the probabilistic methods, Covariance Discriminative Learning (CDL) [5] has been widely used for image set representation. The core idea of CDL method is to represent an image set using a single covariance matrix. One benefit of CDL representation is that it makes no assumption about the set data distribution and thus providing a simple and effective representation for an image set with any kinds of features. However, when data samples are drawn from a union of multiple subspaces, traditional CDL generally fails to provide an accurate and reliable representation.

**Fig 1:**
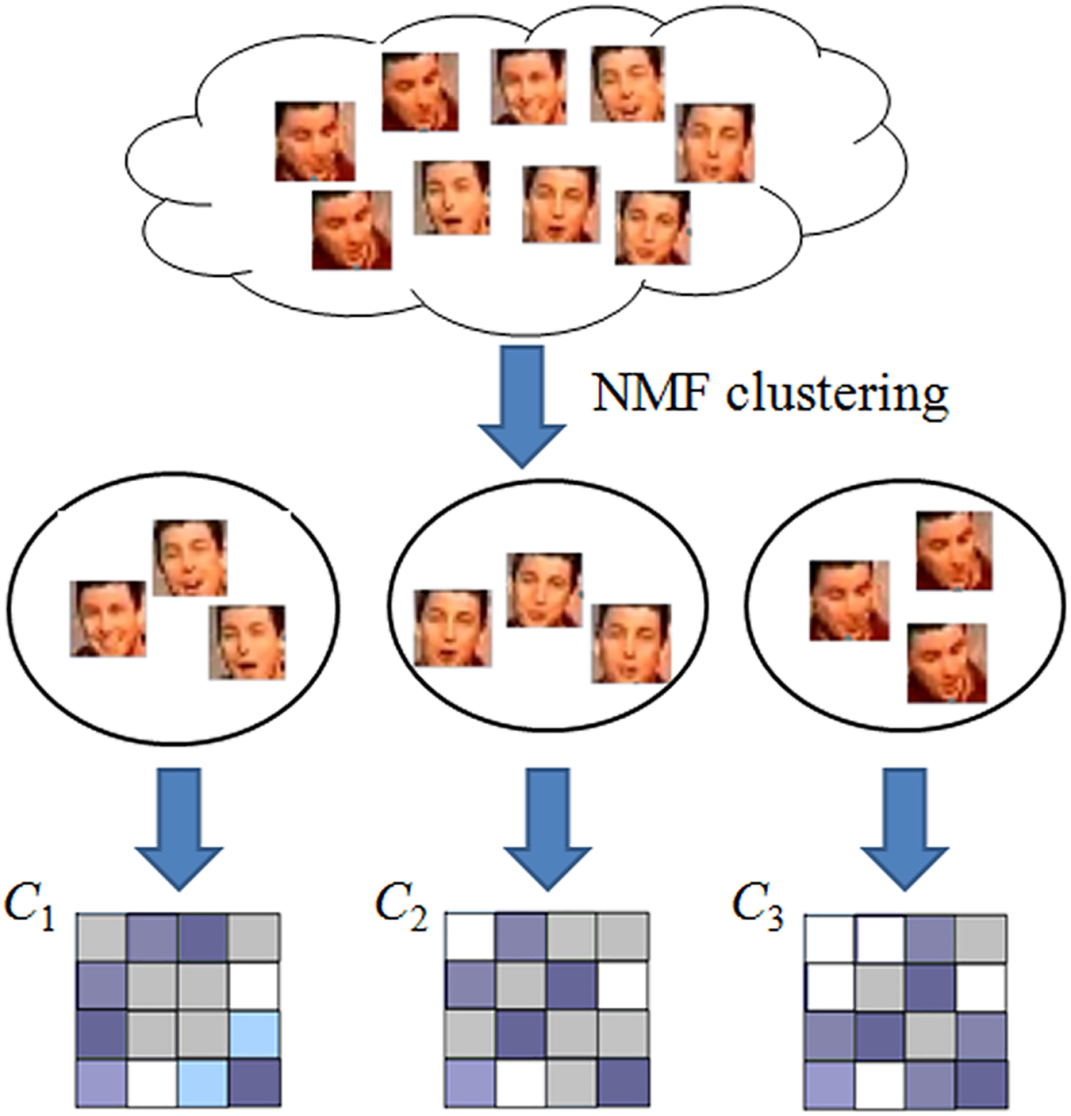
The general flow of our MCDL method.

In this paper, we present a new image set representation method called multiple covariance discriminative learning (MCDL), which aims to represent an image set using multiple covariance matrices with each covariance matrix representing one cluster of images. Comparing with previous single CDL method [5], MCDL explores the data distribution of multiple subspaces more thus providing a more faithful representation. To do that we first use the Nonnegative Matrix Factorization (NMF) technique to cluster the samples into their respective subspaces. Then, we adopt Covariance Discriminative Learning (CDL) on representing each cluster (subset) of images which lies in a single subspace, as shown in Fig 1. Note that covariance-based visual representation has been used in many applications [22, 23]. Different from these works, here we focus on multiple covariance matrices representation, which considers multi-subspaces property of image set data and thus providing a more effective descriptor for image set data. For set classification, we first define a method to measure the similarity between image sets based on MCDL and then adopt KLDA and nearest neighborhood classification method [5] for image set classification. Experimental results on several datasets show the effectiveness and benefits of the proposed MCDL method.

The remainder of this paper is organized as follows. In the materials and methods part, we introduce nonnegative matrix factorization (NMF) data clustering method and propose our Multiple Covariance Matrices representation and Kernel LDA classification method. At last, we apply MCDL method to some datasets to evaluate the effectiveness of the method.

## 2 Materials and methods

The experimental data in our study was acquired legitimately from international standard database, and this study was approved by the Local Ethics Committee of Wuhan University of Technology.

### 2.1 NMF clustering

Nonnegative Matrix Factorization (NMF) [24, 25] is a matrix factorization algorithm that has been widely used in many machine learning problems. Let *X* = (*x*_1_,*x*_2_,…*x*_*n*_) ∈ ℝ^*p*×*n*^ be *n* data points in *p*-dimensional space. The aim of NMF is to find two smaller nonnegative matrices *F* ∈ ℝ^*p*×*k*^ and *G* ∈ ℝ^*n*×*k*^ whose product can approximate the original matrix *X* as close as possible, ie.,

$$X≃FG^{T}.$$ (1)

Using Euclidean distance (or Frobenius norm) residual function, the above approximation problem can be formulated as the following optimization,

$$\min_{F,G}\;\|X-FG^{T}{\|}_{F}^{2}=\sum_{i}(x_{i}-Fg_{i}{)}^{2}$$ (2)

$$s.t.\;F_{ij}\geq 0,\;G_{ij}\geq 0.$$ (3)

From optimization aspect, although the above objective functions are convex in F or G only, there are not convex in both of this two variables. Thus, it is difficult to develop an algorithm to find the global optimal solution for this problem.

Lee and Seung [18] has presented an effective update algorithm which iteratively updates the current solution as follows,

$$F_{ik}\leftarrow F_{ik}\frac{(XG{)}_{ik}}{(FGG^{T}{)}_{ik}},\;G_{ik}\leftarrow G_{ik}\frac{(X^{T}F{)}_{ik}}{(G^{T}F^{T}F{)}_{ik}}$$ (4)

It has been proven that the above update algorithm can converge to a local optimal solution.

The above NMF model has been widely used in many applications. One important aspect of NMF is that it can be used for data clustering. In fact, let $F^{*}=(f_{1}^{*},f_{2}^{*},\cdots f_{k}^{*})$ and G^*^ be the optimal solution of the above optimization problem. Then, $f_{i}^{*},i=1\cdots k$ can be regarded as the cluster centroid, and the optimal *G*_*ik*_ can be viewed as the continuous coefficient of data *x*_*i*_ belonging to cluster *c*_*k*_. In clustering process, we can use the maximum coefficient of *G*_*ik*_ to determine the cluster label of data *x*_*i*_.

### 2.2 Image set modeling with multiple covariance matrices

In this section, an effective method is proposed to represent image sets by using multiple covariance matrices. Based on this representation, a similarity metric between two image sets is further computed.

#### A. Image set representation

We first propose an effective image set representation by using multiple covariance matrices, called Multiple Covariance Discriminative Learning (MCDL). Formally, given a video (or image set) $X=(x_{1},x_{2},\cdots x_{n})$, we first use the above NMF method to do clustering on X and obtain clustering results $X=\{X_{1},X_{2},\cdots X_{k}\}$ with *k* clusters. Here, *X*_*i*_ is the image subset belonging to the *i*-th cluster.

Then, each cluster *X*_*i*_ is represented with a *d* × *d* covariance matrix as follows,

$$C_{i}=\frac{1}{n-1}\sum_{h=1}^{p}(X_{i}(h)-{\bar{X}}_{i})(X_{i}(h)-{\bar{X}}_{i}{)}^{T}.$$ (5)

where ${\bar{X}}_{i}$ is the mean of the *i*-th cluster, and *X*_*i*_(*h*) is the *h*-th element in cluster *X*_*i*_.

At last, the whole image set *X* can be represented by using a set of covariance matrices as follows,

$$C=\{C_{1},C_{2},\cdots C_{k}\}.$$ (6)

when *k* = 1, our MCDL degenerates to the traditional CDL method [5]. Therefore, the proposed MCDL can be regarded as a general extension of CDL representation. Comparing with CDL, MCDL can represent the variations of images in an image set more sufficiently and effectively while maintaining the benefit of CDL representation.

#### B. Similarity metric for MCDL

Based on the above MCDL representation, we propose a method to define a similarity metric between two image sets *S*_1_ and *S*_2_ whose covariance matrix representations are $C^{1}$ and $C^{2}$ respectively. Formally, let $C^{1}=\{C_{1}^{1},C_{2}^{1},\cdots C_{k}^{1}\}$ and $C^{2}=\{C_{1}^{2},C_{2}^{2},\cdots C_{k}^{2}\}$, it is known that for any covariance matrix $C_{h}^{1}$ or $C_{l}^{2}$, it is symmetric positive definite (SPD). For any SPD matrix, it does not lie in a Euclidean space but on the Riemannian manifold [5, 26]. Therefore, it is necessary to map $C_{h}^{1}$ or $C_{k}^{2}$ from Riemannian manifold to Euclidean space using the following logarithm operator [5],

$$\Psi_{M\to E}:\;C⟶\log\;(C)$$ (7)

where

$$\log\;(C)=\{\log\;(C_{1}),\log\;(C_{2}),\cdots \log\;(C_{k})\}.$$

Using this mapping $\Psi_{M\to E}$, the similarity between two image sets *S*_1_ and *S*_2_ can be defined as the following three main steps.

Step 1. Compute the similarity between covariance matrices $C_{i}^{1}$ and $C_{j}^{2}$ as the inner product between them, i.e.,

$$K_{ij}=k(C_{i}^{1},C_{j}^{2})=Tr(\log\;(C_{i}^{1})\cdot \log\;(C_{j}^{2}))$$ (8)

where *Tr*(*A*) is the trace norm function of matrix *A*.

Step 2. Compute the optimal mapping *f* between two covariance matrix set $C^{1}$ and $C^{2}$ by solving the following optimization

$$\min_{f}\;\sum_{i=1}^{k}\sum_{j=1}^{k}f_{ij}\;K_{ij}$$

$$s.t.\;\sum_{i}f_{ij}=1,\sum_{j}f_{ij}=1,f_{ij}\in \{0,1\}$$ (9)

The above problem is known as bipartite graph matching problem and can be efficiently and effectively solved by using Hungarian algorithm.

Step 3. Calculate the mapping similarity between covariance matrix set $C^{1}$ and $C^{2}$ as follows,

$$K(C^{1},C^{2})=\sum_{i=1}^{k}Tr(\log\;(C_{i}^{1})\cdot \log\;(C_{f(i)}^{2}))$$ (10)

Note that the above similarity function K is the combination of linear kernel functions $k(C_{i}^{1},C_{j}^{2})$. Therefore it is also a desired kernel function.

### 2.3 Image set classification

Based on the above MCDL representation and associated metric definition method, we can provide an effective classification method for image set. Generally, our classification method contains two main steps. Firstly, we use the Kernel Linear Discriminant Analysis (KLDA) [27, 5] method to extract a kind of discriminative feature for our MCDL representation. Then, we use nearest neighbor classification method to do classification on image set data. Let $X=\{X_{1},X_{2},\cdots ,X_{m}\}$ be *m* image sets belonging to *c* classes. For each pair of set $X_{i}$ and $X_{j}$, we extract the MCDL representations for them and then compute the similarity kernel function K(i,j) between them. The aim of KLDA is to solve the following optimization,

$$\max_{p}\;\frac{tr\left(p^{T}KLKp\right)}{p^{T}KKp}\;s.t.\;p^{T}p=1$$ (11)

where K is the kernel matrix which is computed using Eq (10), and *L* is the class label matrix defined as,

$$L_{ij}=\{\begin{array}{ll} \frac{1}{m_{k}} & \mathrm{if}\;X_{i},\;X_{j}\;\mathrm{belong}\;\mathrm{to}\;\mathrm{the}\;k-\mathrm{th}\;\mathrm{class}\\ 0 & \mathrm{otherwise}. \end{array}$$ (12)

where *m*_*k*_ is the number of data points belonging to class *k* and $\Sigma_{k=1}^{c}m_{k}=m$. It is well known that optimal solution *p* can be obtained by computing the eigenvector corresponding to the largest eigenvalue. By further grouping the first largest (*c* − 1) eigenvectors, we can obtain *P* = [*p*_1_,⋯,*p*_*c*−1_] and get the *c* − 1 projected feature vector by $z_{i}=P^{T}K_{i\cdot }$ where $K_{i\cdot }=[K_{1i},\cdots ,K_{mi}{]}^{T}$. After KLDA projection, we then use nearest neighbor classification to classify image sets [5].

## 3 Experiments and results

In this section, we implement and test our MCDL method on several datasets to evaluate the effectiveness and benefits of our method. The detail introduction of these datasets are given below. These datasets have been widely used in many other methods. We have compared our MCDL method with some other methods including traditional Covariance Discriminative Learning (CDL) [5], Set to Set Distance Metric Learning (SSDML) [28], Manifold Discriminant Analysis (MDA) [21], Manifold-Manifold Distance (MMD) [20] and Discriminant Canonical Correlations [19].

### 3.1 Datasets description

- CMU MoBo [29] dataset contains 96 sequences of 24 persons. Each video contains approximately 300 frames.
- YTC [30] dataset contains 1910 face videos of 47 subjects. Each video contains several hundreds of frames.
- Cambridge-Gesture [31] dataset has 900 video sequences of 9 gestures in whole. Each gesture contains 100 videos. We divide it into five sets.
- ETH-80 [32] dataset contains 8 object categories in whole. Each category contains 10 object subcategories.

All the images used in these four datasets have been resized to the same 20 × 20 intensity images to make the same consistent dimension.

### 3.2 Classification results

**Fig 2:**
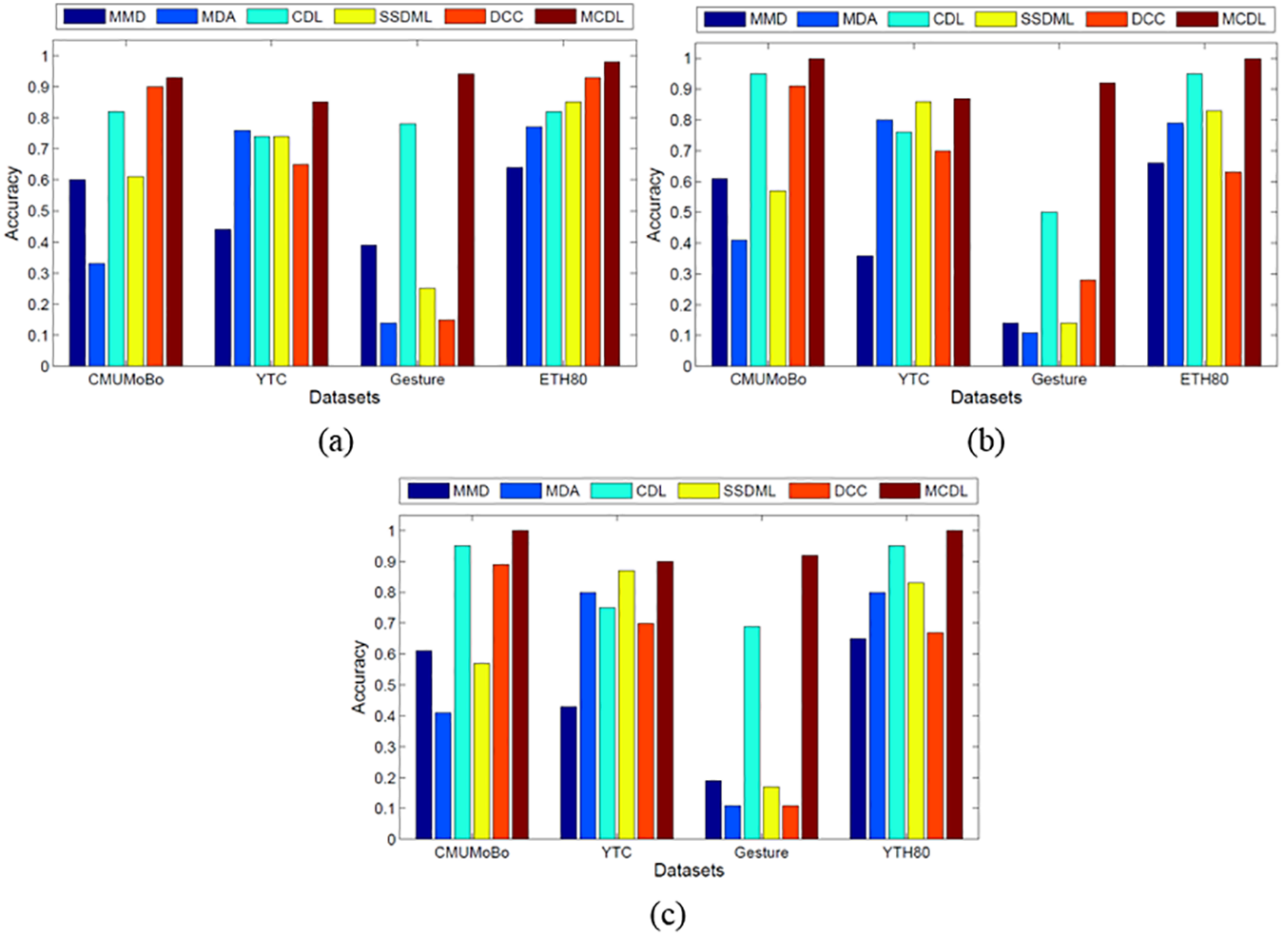
Classification accuracies of different methods on different datasets. (a) Average results on 50% sampling. (b) Average results on 70% sampling. (c) Average results on 90% sampling.

We conduct the classification experiments for three cases by randomly selecting 50%, 70%, 90% image sets respectively for gallery and the rest image sets for probe. For fair comparison, the important parameters of each method were empirically tuned according to the recommendations in the original references. Fig 2 shows the classification results of all methods on the four datasets.

Here, we can observe that (1) CDL can return better performance in general, which indicates the effectiveness and benefits of the CDL method on conducting image set classification tasks. (2) MCDL obtains obvious better performance than other methods on gesture. Because in this dataset, the image in each set are usually lied on multiple subspaces. As discussed before, MCDL method performs more effectively and suitably for the data lying on multiple subspaces because it uses multiple covariance matrices representation instead of traditional single model representations such as MMD, MDA and DCC. (3) MCDL outperforms traditional CDL method and obtains the best performance on the four datasets. This clearly indicates the robustness and effectiveness of the proposed MCDL method on conducting image set representation and classification task. (4) MCDL obviously outperforms CDL on Gesture dataset. For this dataset, the variations of images in each set are very large due to different gestures and thus can be divided into several clusters. In this case, the proposed MCDL can capture these variations more effectively and sufficiently than single CDL.

We also test our method on the standard setting, which is summarized as follows. For each person in CMU MoBo [29] dataset, one set is used for gallery and the rest for probe. In YTC [30] dataset, we randomly chose 3 sets for gallery and 6 sets for probe. For Cambridge-Gesture [31] dataset, the first set for gallery and the rest four sets are used for probe. For each category in ETH-80 [32] dataset, five objects are selected for gallery and the rest 5 objects for probe. The results are summarized in Table 1. It can be seen that our method can return better performance than other compared methods, which further demonstrates the robustness of the proposed MCDL method on image set classification tasks.

**Table 1:** Average accuracies of different methods on four datasets.

Methods	CMUMoBo	YTC	Gesture	ETH-80
MMD	0.90	0.63	0.10	0.86
MDA	0.94	0.65	0.11	0.89
CDL	0.94	0.70	0.69	0.97
SSDML	0.24	0.82	0.17	0.75
DCC	0.88	0.65	0.15	0.91
**MCDL**	**0.92**	**0.83**	**0.94**	**0.98**

The classification accuracy of different methods on four datasets are summarized in Table 1. Here, we can note that comparing with other methods, CDL can return better performance, which indicates the effectiveness and benefits of the CDL method. Our MCDL generally outperforms traditional CDL method and obtains the best performance. This clearly indicates the robustness and effectiveness of the proposed MCDL method on conducting image set representation and classification task.

## 4 Conclusion

In this paper, we present a new image set representation method called multiple covariance discriminative learning (MCDL). The aim of MCDL is to represent an image set using multiple covariance matrices and each covariance matrix represents one cluster of images. To do that, firstly we use a Nonnegative Matrix Factorization (NMF) to conduct image clustering within each image set. Then, we adopt Covariance Discriminative Learning (CDL) to represent each cluster (subset) of images. In terms of classification, we first define a method to measure the similarity between image sets based on MCDL and then adopt KLDA and nearest neighborhood classification method for image set classification. Experimental results show the effectiveness and benefits of the proposed method.

## Supporting information

S1 Fig The general flow of our MCDL method. (TIF)

S2 Fig Classification accuracies of different methods on different datasets. (TIF)

S1 Table Average accuracies of different methods on four datasets. (DOCX)
