# Supplementary figures and images for: Video based object representation and classification using multiple covariance matrices
Source: PLoS One. 2017 Jun 8;12(6):e0176598. doi: 10.1371/journal.pone.0176598 (PMC5464534; doi:10.1371/journal.pone.0176598)

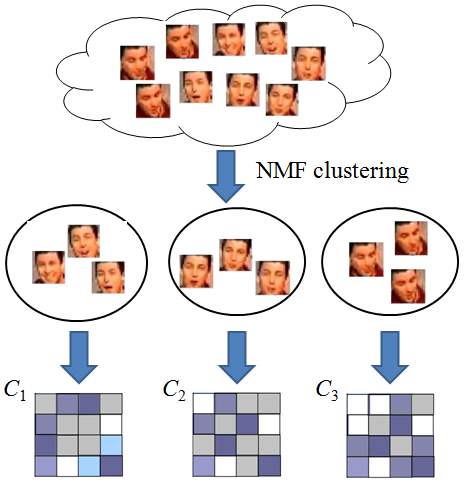

Supplement: S1 Fig — (TIF) [file pone.0176598.s001.tif]

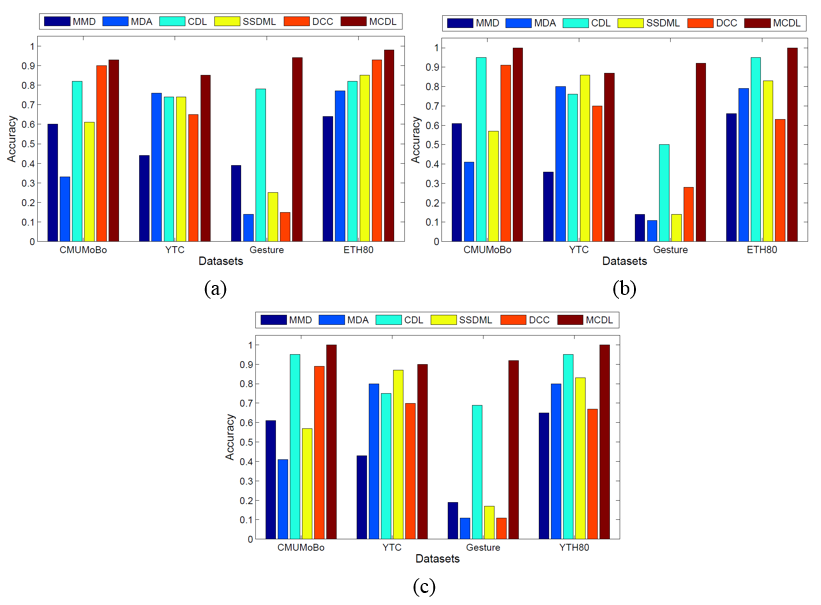

Supplement: S2 Fig — (TIF) [file pone.0176598.s002.tif]
