# Supplementary material for: Video based object representation and classification using multiple covariance matrices
Source: PLoS One. 2017 Jun 8;12(6):e0176598. doi: 10.1371/journal.pone.0176598 (PMC5464534; doi:10.1371/journal.pone.0176598)
Supplement: S1 Table — (DOCX) [file pone.0176598.s003.docx]

Table 1 Average accuracies of different methods on four datasets.

| Methods | CMUMoBo | YTC | Gesture | ETH-80 |
| --- | --- | --- | --- | --- |
| MMD | 0.90 | 0.63 | 0.10 | 0.86 |
| MDA | 0.94 | 0.65 | 0.11 | 0.89 |
| CDL | 0.94 | 0.70 | 0.69 | 0.97 |
| SSDML | 0.24 | 0.82 | 0.17 | 0.75 |
| DCC | 0.88 | 0.65 | 0.15 | 0.91 |
| **MCDL** | **0.92** | **0.83** | **0.94** | **0.98** |
